# Novel Insights into FH-associated Disease are KEAPing the Lid on Oncogenic HIF Signalling

**DOI:** 10.18632/oncotarget.348

**Published:** 2011-11-06

**Authors:** Julie Adam, Peter J. Ratcliffe, Patrick J. Pollard

**Affiliations:** ^1^ The Nuffield Department of Medicine, University of Oxford, Roosevelt Drive, Oxford, OX3 7BN, UK

Fumarate hydratase (FH) encodes a Krebs cycle enzyme which is mutated in hereditary leiomyomatosis and renal cell cancer (HLRCC). Fumarate is a potent inhibitor of a class of enzymes, 2-oxoglutarate (2OG) oxygenases, that require oxygen, non-haem Fe(II), and 2OG for activity. Importantly, loss of FH function has been shown to result in elevated levels of fumarate in cells and tumors. Recent work has demonstrated that fumarate-mediated inhibition of a sub-class of 2OG oxygenases, the hypoxia-inducible factor prolyl hydroxylase enzymes (HIF-PHDs), causes stabilisation of HIF and the upregulation of a broad range of HIF-target genes, including those that stimulate cell growth and angiogenesis, which may in turn contribute to tumorigenesis [1]. Previously we generated a conditional *Fh1* (the ortholog of human FH) knockout mouse and showed that kidney-specific deletion of Fh1 recapitulated important aspects of the human disease, including activation of HIF and the development of hyperplastic renal cysts [2]. HIF dysregulation following inactivation of the *FH* tumor suppressor gene has therefore been hypothesised as a plausible mechanism for HLRCC tumorigenesis.

In addition to inhibiting 2OG oxygenases, fumarate modifies cysteine residues in multiple proteins to form S-(2-succinyl)-cysteine (2SC) and this chemical modification, termed succination, can have important functional consequences, such as the inactivation of glyceraldehyde-3-phosphate dehydrogenase in both an *in vivo* and *in vitro* setting [3]. Of potential clinical importance, we have reported recently that FH-deficient cells and tumors specifically exhibit high levels of 2SC that are absent in normal cells and tissues, thus providing a potential diagnostic biomarker and an alternative candidate or parallel mechanism for tumorigenesis [4].

In last month's edition of Cancer Cell, we demonstrate through multiple genetic crosses in mice, that neither the presence of *Hif*, nor the absence of *Phds*, are required for renal cyst formation in an Fh1-deficient background and that surprisingly, loss of *Hif-1α* actually exacerbates the cystic phenotype [5]. Furthermore, we show that murine Fh1 deficient renal cysts, mouse embryonic fibroblasts and human FH-deficient cells and tissues exhibit a striking upregulation of the antioxidant signalling pathway mediated through Nuclear factor (erythroid-derived 2)-like 2 (NRF2) as a direct consequence of FH inactivation and independent of HIF/PHD signalling [5].

NRF2 has been identified as a master regulatory molecule controlling the adaptive response of cells to oxidative and electrophilic stress by interaction and activation of multiple NRF2 target genes that contain antioxidant response elements [6]. Cellular levels of NRF2 are controlled by Kelch-like ECH-associated protein 1 (KEAP1). In our work and the accompanying manuscript by Ooi and colleagues it is shown by tandem mass spectrometry that fumarate modifies critical cysteine residues (Cys155 and Cys288) within KEAP1 by succination, abrogating its ability to ameliorate antioxidant signaling [5, 7].

We therefore postulate that loss of FH leads to an accumulation of fumarate that modifies key cysteine residues in KEAP1 preventing it from binding NRF2. We propose that dysregulation of NRF2, rather than activation HIF, may be an alternative candidate pathway in tumorigenesis associated with loss of FH.

Studies of HLRCC, resulting from the loss of FH in cells, have revealed a complex interplay between genetic mutation and dysregulation of cellular metabolism. Given the resurgence of interest in the metabolic switch associated with cancer cells proposed many years ago by Warburg, HLRCC affords a new paradigm to investigate the importance of alterations in cellular metabolism and cancer.

## References

[1] Isaacs JS, Jung YJ, Mole DR, Lee S, Torres-Cabala C, Chung YL, Merino M, Trepel J, Zbar B, Toro J, Ratcliffe PJ, Linehan WM, Neckers L (2005). HIF overexpression correlates with biallelic loss of fumarate hydratase in renal cancer: novel role of fumarate in regulation of HIF stability. Cancer Cell.

[2] Pollard PJ, Spencer-Dene B, Shukla D, Howarth K, Nye E, El-Bahrawy M, Deheragoda M, Joannou M, McDonald S, Martin A, Igarashi P, Varsani-Brown S, Rosewell I, Poulsom R, Maxwell P, Stamp GW (2007). Targeted inactivation of fh1 causes proliferative renal cyst development and activation of the hypoxia pathway. Cancer Cell.

[3] Blatnik M, Thorpe SR, Baynes JW (2008). Succination of proteins by fumarate: mechanism of inactivation of glyceraldehyde-3-phosphate dehydrogenase in diabetes. Ann N Y Acad Sci.

[4] Bardella C, El-Bahrawy M, Frizzell N, Adam J, Ternette N, Hatipoglu E, Howarth K, O'Flaherty L, Roberts I, Turner G, Taylor J, Giaslakiotis K, Macaulay VM, Harris AL, Chandra A, Lehtonen HJ (2011). Aberrant succination of proteins in fumarate hydratase-deficient mice and HLRCC patients is a robust biomarker of mutation status. J Pathol.

[5] Adam J, Hatipoglu E, O'Flaherty L, Ternette N, Sahgal N, Lockstone H, Baban D, Nye E, Stamp GW, Wolhuter K, Stevens M, Fischer R, Carmeliet P, Maxwell PH, Pugh CW, Frizzell N (2011). Renal Cyst Formation in Fh1-Deficient Mice Is Independent of the Hif/Phd Pathway: Roles for Fumarate in KEAP1 Succination and Nrf2 Signaling. Cancer Cell.

[6] Hayes JD, McMahon M (2009). NRF2 and KEAP1 mutations: permanent activation of an adaptive response in cancer. Trends Biochem Sci.

[7] Ooi A, Wong JC, Petillo D, Roossien D, Perrier-Trudova V, Whitten D, Min BW, Tan MH, Zhang Z, Yang XJ, Zhou M, Gardie B, Molinie V, Richard S, Tan PH, Teh BT (2011). An Antioxidant Response Phenotype Shared between Hereditary and Sporadic Type 2 Papillary Renal Cell Carcinoma. Cancer Cell.

